# Longitudinal effects of social media feedback on psychological distress in young adults: the mediating roles of self-esteem and social comparison

**DOI:** 10.1186/s40359-026-04912-9

**Published:** 2026-06-05

**Authors:** Jinglun Wu, Zhaomo Zhang

**Affiliations:** 1 Basic Department, Beijing Jingbei Vocational College, Beijing, 101400 China; 2 School of Arts and Design, Sanming University, Sanming, 365000 China

**Keywords:** Social media feedback, Psychological distress, Self-esteem, Social comparison, Longitudinal study

## Abstract

**Background:**

Social media feedback, particularly likes and comments, has become an important source of social validation for young adults. However, the longitudinal effects of such feedback on psychological distress and the underlying psychological mechanisms remain unclear.

**Methods:**

This study employed a three-wave longitudinal design over eight months to examine the effects of social media likes and comments feedback on depressive and anxiety symptoms among 398 Chinese young adults (mean age = 21.34 years, 58.3% female). Cross-lagged panel models were used to test bidirectional relationships, and bias-corrected bootstrap methods were applied to examine the mediating roles of self-esteem and social comparison tendency.

**Results:**

Lower frequency of like feedback at baseline significantly predicted higher levels of depressive (β = −0.14, *p* < 0.01) and anxiety (β = −0.11, *p* < 0.05) symptoms at four-month follow-up. Reverse paths were non-significant. Self-esteem and social comparison tendency mediated these relationships, together explaining 65.0% of the total effect, with self-esteem accounting for 37.1% and social comparison for 27.9%.

**Conclusions:**

Social media feedback longitudinally influences psychological distress among young adults primarily through self-evaluation mechanisms. Interventions targeting self-esteem resilience and healthy social comparison strategies may help mitigate negative psychological effects of social media use.

## Introduction

### Social media feedback and mental health

Social media has become deeply integrated into the daily lives of contemporary young adults, with the usage rate among Chinese individuals aged 18 to 29 exceeding 95% (China Internet Network Information Center, 2024). While these platforms serve multiple functions, one of their most psychologically significant features is the provision of social feedback through interactive mechanisms such as likes and comments. These feedback signals provide users with immediate and quantifiable indicators of how their shared content is received by others. These digitized indicators of social approval have gradually become important references for young users to evaluate their social worth and interpersonal attractiveness. Empirical evidence supports this observation: Burrow and Rainone (2017) [1] demonstrated experimentally that the number of likes received significantly influenced participants’ momentary self-worth, and Chua and Chang (2016) [2] found through qualitative interviews that adolescents commonly used like counts as a metric for gauging their social popularity and likability among peers. Concurrently, the mental health status of young populations has received increasing attention. Epidemiological survey data indicate that the detection rates of depressive and anxiety symptoms among Chinese college students have shown an upward trend in recent years [3, 4]. The etiology of this phenomenon is complex and multifactorial, and the association between social media use and mental health has become a focal issue of concern for both academia and the public.

Before reviewing the relevant literature, it is necessary to clarify the conceptual definition and classification of social media feedback. In this study, social media feedback is defined as the observable and quantifiable interactive responses that users receive from others in their social network after publishing content on social media platforms. These feedback signals serve as direct indicators of how one’s content is received, recognized, and valued within one’s digital social environment.

Social media feedback can be broadly classified into two categories based on the nature of the response: paralinguistic digital affordances and verbal feedback [5]. Likes represent the most prototypical form of paralinguistic digital affordances—they are standardized, one-click expressions of approval that require minimal cognitive effort from the sender. Comments, in contrast, constitute verbal feedback that involves higher cognitive investment in composing specific linguistic content, thereby conveying richer semantic information including particular attitudes, emotions, and evaluations.

Several key dimensions differentiate these two forms of feedback in terms of their potential psychological impact. First, regarding interaction cost, likes are virtually effortless, whereas comments demand greater time and cognitive resources, which may lead recipients to perceive comments as signals of deeper social investment [6]. Second, regarding information richness, likes provide a unidimensional binary signal of approval (present or absent), while comments contain multidimensional semantic content that can range from supportive to critical. Third, and perhaps most importantly for the present study, regarding quantifiability and comparability, like counts are displayed as clear numerical indicators that are highly amenable to social comparison across users, whereas the value of comments is more difficult to quantify and compare. These differences suggest that likes and comments may exert their psychological effects through partially distinct pathways—likes may more strongly trigger social comparison processes due to their high quantifiability, while comments may influence self-evaluation more through their specific content. Given these theoretical distinctions, the present study examines likes and comments as two separate dimensions of social media feedback rather than treating them as a unitary construct.

Research examining the relationship between social media use and mental health has accumulated rapidly in recent years, though findings exhibit considerable heterogeneity [7, 8]. Early studies focused primarily on overall usage duration, finding small to moderate associations with depression and anxiety symptoms [9, 10], but with highly variable effect sizes across studies [11]. Scholars have increasingly recognized that specific forms and content of social media use may be more predictive of mental health outcomes than mere usage duration [12], prompting closer examination of particular interaction characteristics—especially feedback mechanisms such as likes and comments. Nesi and Prinstein’s research demonstrated that adolescents’ feedback-seeking behaviors on social media were significantly positively correlated with depressive symptoms, and individuals who received less positive feedback exhibited higher levels of psychological distress [13]. Burrow and Rainone’s experimental study found that participants who received more likes reported higher self-worth and more positive emotional states, suggesting that social media feedback may directly impact individuals’ immediate psychological experiences [1]. A systematic review focusing on Instagram use indicated consistent positive associations between platform use and social comparison, body dissatisfaction, and eating disorder symptoms, with these associations largely attributable to the visual and quantifiable characteristics of the platform that facilitate social comparison processes [14, 15].

Although the aforementioned studies have provided important evidence for understanding the association between social media feedback and mental health, the existing literature exhibits notable limitations in several respects. Regarding research design, the vast majority of studies have employed cross-sectional designs, which preclude the determination of temporal relationships and causal directions between variables [16]. The core question of whether social media feedback is a cause or consequence of mental health problems remains inadequately addressed. Regarding mechanisms of action, the internal psychological processes through which social media feedback affects mental health remain unclear. Although some studies have proposed theoretical hypotheses, empirical tests of mediating mechanisms are relatively scarce, particularly lacking systematic examination of multiple psychological mechanisms within longitudinal frameworks [17]. Regarding research populations, existing studies have predominantly focused on Western adolescent samples, with relatively limited empirical research on Chinese young adult populations [18, 19]. Given that cultural context can shape individuals’ sensitivity to social evaluative feedback, validating relevant theoretical hypotheses within the Chinese context holds particular academic value and practical implications. Research in cultural psychology has established that individuals in collectivist cultures tend to place greater emphasis on social harmony, group belonging, and others’ evaluations of the self compared to those in individualist cultures [20]. In the Chinese cultural context specifically, the concept of “face” (mianzi)—referring to one’s social reputation and public image—makes social approval signals particularly salient for self-evaluation [21]. These cultural characteristics suggest that Chinese young adults may be especially sensitive to quantified social feedback such as likes and comments on social media, yet the majority of existing research has been conducted with Western samples [18], leaving this possibility largely untested.

### The mediating role of self-esteem: a sociometer theory perspective

Sociometer theory provides an important theoretical perspective for understanding the mechanisms through which social media feedback influences mental health. Proposed by Leary and colleagues [22], the core proposition of this theory holds that self-esteem is not a stable personality trait but rather serves as an internal psychological indicator through which individuals monitor their degree of social acceptance, continuously and dynamically adjusting in response to signals of acceptance or rejection from the social environment. When individuals perceive acceptance, recognition, and valuation from others, self-esteem levels correspondingly increase; conversely, when encountering social rejection or neglect, self-esteem levels decline. In the context of social media, likes and positive comments can be viewed as digitized signals of social acceptance and approval. Higher frequencies of positive feedback convey messages such as “you are liked, attended to, and valued,” thereby maintaining or enhancing individuals’ self-esteem levels; whereas lower frequencies of feedback may be interpreted as signals of social neglect or value depreciation, leading to diminished self-esteem [23]. Extensive empirical research has confirmed robust negative associations between self-esteem and negative emotions such as depression and anxiety, with low self-esteem serving both as a risk factor for and core symptomatic manifestation of depression and anxiety [24]. Based on this theoretical logic, it can be inferred that self-esteem may mediate the relationship between social media feedback and depressive and anxiety symptoms, wherein lower feedback frequency leads to decreased self-esteem levels, which in turn increases the risk of depression and anxiety symptoms. The social media self-esteem susceptibility model proposed by Valkenburg and colleagues further supports this inference, emphasizing that social media feedback has immediate and significant effects on adolescent self-esteem, and that fluctuations in self-esteem are closely associated with subsequent emotional health [25–27].

### The mediating role of social comparison

Social comparison theory provides another important framework for understanding the psychological effects of social media feedback. This classic theory, proposed by Festinger in 1954 [28], posits that individuals possess an intrinsic drive to evaluate their own abilities and opinions, and when objective standards are unavailable, people tend to achieve self-evaluation through comparison with others. Social comparison can be categorized into upward comparison (comparing oneself to others perceived as superior) and downward comparison (comparing oneself to others perceived as inferior), with upward comparison typically associated with negative emotional experiences [29]. The unique design features of social media platforms—particularly the highly visual and quantifiable presentation of social indicators such as like counts, comment counts, and follower counts—create ubiquitous opportunities and cues for social comparison among users [30]. When individuals observe that their posted content receives far fewer likes than others in their social network, such upward social comparison may trigger feelings of relative deprivation, envy, and devaluation of self-worth [23]. Moreton and Greenfeld’s qualitative study of British university students profoundly revealed this psychological process, with interviewees commonly reporting that they associated like counts with their own popularity and likability, experiencing notably negative emotional experiences when receiving fewer likes than expected or fewer than others [15]. Faelens and colleagues’ systematic review of the relationship between Instagram use and mental health also indicated that social comparison is one of the core mechanisms explaining the association between social media use and psychological distress [14]. Therefore, social comparison tendency may constitute another mediating pathway through which social media feedback influences mental health.

### The present study

Synthesizing the aforementioned theoretical analyses and literature review, the present study aims to systematically examine the longitudinal effects of social media likes and comments feedback on depressive and anxiety symptoms among Chinese young adults through a three-wave longitudinal tracking design, and to test the dual mediating roles of self-esteem and social comparison in this relationship. Compared with existing research, the innovations of this study are reflected in the following aspects: adopting a longitudinal tracking design and testing bidirectional relationships to provide stronger evidence for the temporal directionality of the relationship between social media feedback and mental health [16, 31]; integrating sociometer theory and social comparison theory to simultaneously test two mediating pathways within a unified analytical framework, thereby revealing the multiple psychological mechanisms through which social media feedback affects mental health; distinguishing between likes and comments as two types of feedback to examine their differential effects on mental health; and using Chinese young adults as research participants to provide empirical data for cross-cultural validation of relevant theories.

Based on the above theoretical foundation and research objectives, this study proposes the following hypotheses, organized into two levels: direct longitudinal effects and mediating mechanisms.

At the first level, we hypothesize that social media feedback at T1 will longitudinally predict mental health outcomes at T2, after controlling for baseline levels of the outcome variables:

- H1a: Lower frequency of like feedback at T1 will predict higher levels of depressive symptoms at T2.
- H1b: Lower frequency of like feedback at T1 will predict higher levels of anxiety symptoms at T2.
- H2a: Lower frequency of comment feedback at T1 will predict higher levels of depressive symptoms at T2.
- H2b: Lower frequency of comment feedback at T1 will predict higher levels of anxiety symptoms at T2.

Although H1 and H2 predict effects in the same direction, they are specified separately because likes and comments differ substantially in interaction cost, information richness, and quantifiability (as discussed in Sect. Social media feedback and mental health), which may result in differential effect sizes.

At the second level, we hypothesize that the longitudinal effects of social media feedback on mental health will be transmitted through self-evaluative mechanisms, following the temporal sequence of T1 (predictor) → T2 (mediator) → T3 (outcome):

- H3: Self-esteem at T2 will mediate the longitudinal relationship between social media feedback at T1 and depressive and anxiety symptoms at T3. Specifically, lower feedback frequency will predict lower self-esteem, which in turn will predict higher levels of depressive and anxiety symptoms.
- H4: Social comparison tendency at T2 will mediate the longitudinal relationship between social media feedback at T1 and depressive and anxiety symptoms at T3. Specifically, lower feedback frequency will predict greater social comparison tendency, which in turn will predict higher levels of depressive and anxiety symptoms.

## Methods

### Participants

This study employed a longitudinal tracking design and conducted data collection at three comprehensive universities in the capital city of Fujian Province, China, from January 2025 to September 2025. Sample recruitment was conducted through a combination of online questionnaire platforms and offline promotion, specifically including posting recruitment information on official social media accounts of each university, displaying recruitment posters on campus bulletin boards, and distributing questionnaire links to student groups through academic advisors. To ensure that the sample size met the statistical power requirements for longitudinal mediation model testing, the research team conducted a priori power analysis using G*Power 3.1 software prior to data collection. Based on the expected small to moderate effect size (f² = 0.10) in cross-lagged panel models, significance level (α = 0.05), desired statistical power (1 − β = 0.80), and an estimated 20% sample attrition rate, the required initial sample size was calculated to be 450 participants.

The inclusion criteria for participants were established as follows: enrolled university students aged between 18 and 30 years; daily use of at least one social media platform for more than 30 min; posting content on social media (including images, videos, or status updates) at least once per week; ability to independently complete questionnaires in Chinese and voluntary participation in the study. Exclusion criteria included: individuals currently receiving psychiatric treatment or psychological counseling; individuals diagnosed with depression, anxiety disorder, or other psychiatric disorders within the past six months; individuals unable to commit to completing data collection at all three time points. Following eligibility screening, 486 university students met the inclusion criteria and completed the baseline (T1) survey. At the second time point (T2, four-month interval), 421 participants completed the measurement, representing a loss of 65 participants (attrition rate of 13.4% from T1 to T2). At the third time point (T3, eight-month interval), 398 participants completed the measurement, representing a further loss of 23 participants (attrition rate of 5.5% from T2 to T3). The overall retention rate across the full study period was 81.9%. To systematically assess the potential impact of sample attrition on research results, independent samples t-tests and chi-square tests were conducted to compare baseline demographic characteristics and core variables between participants who completed all three measurements and those who withdrew. Results showed no significant differences between the two groups in age, gender, social media usage duration, depression and anxiety levels (all *p* > 0.05), indicating that sample attrition was random and would not produce systematic bias in the validity of research conclusions.

The demographic characteristics of the 398 participants who completed all three data collection waves are detailed in Table 1. The mean age of the sample was 21.34 years (SD = 2.18), with an age range of 18 to 28 years. Females comprised 58.3% (*n* = 232) and males comprised 41.7% (*n* = 166) of the sample. Regarding academic major distribution, humanities and social sciences accounted for 42.7%, science and engineering for 38.4%, and medicine and other fields for 18.9%. Participants reported an average daily social media usage time of 3.26 h (SD = 1.54). The most frequently used platforms were WeChat (89.2%), Weibo (76.4%), Douyin (71.8%), and Xiaohongshu (54.3%).

**Table 1:** Demographic characteristics of participants (*N* = 398)

Variable	*n*/M	%/SD
Age (years)	21.34	2.18
Age range	18–28	—
Gender		
Male	166	41.7%
Female	232	58.3%
Academic Major		
Humanities and Social Sciences	170	42.7%
Science and Engineering	153	38.4%
Medicine and Others	75	18.9%
Daily Social Media Use (hours)	3.26	1.54
Most Frequently Used Platforms^a^		
WeChat	355	89.2%
Weibo	304	76.4%
Douyin (TikTok)	286	71.8%
Xiaohongshu (RED)	216	54.3%

^a^ Percentages sum to more than 100% because participants could select multiple platforms

The data presented in Table 1 indicate that the gender ratio and age distribution of this study’s sample are generally consistent with the overall characteristics of the Chinese college student population, and the social media usage patterns conform to the typical behavioral characteristics of contemporary young users [19, 31], providing a foundation for generalizing the research conclusions to broader young adult populations.

### Procedure

This study strictly adhered to the ethical principles of the Declaration of Helsinki. The research protocol was formally approved by the Institutional Ethics Review Committee of the affiliated university prior to the initiation of data collection (Ethics Approval Number: SMU-IRB-2025-013). All participants were required to read an informed consent document before completing the questionnaire. This document provided detailed explanations of the research purpose, participation procedures, potential risks and benefits, data confidentiality measures, and the right to withdraw from the study at any time. Participants indicated informed consent by checking an online confirmation box.

Data collection was conducted through online questionnaire surveys implemented via the Wenjuanxing platform. The entire longitudinal tracking study comprised three measurement time points, with approximately four-month intervals between each. This interval setting was determined with reference to methodological literature on previous longitudinal studies examining the relationship between social media use and mental health [16, 31], balancing the need to capture dynamic changes between variables while avoiding repeated measurement effects due to intervals that are too short. At each data collection time point, the research team sent questionnaire links through contact information provided by participants (mobile phone numbers or email addresses) and sent two reminder notifications within one week to improve questionnaire response rates. To acknowledge participants’ time and effort, each completed questionnaire was rewarded with an electronic shopping voucher worth 15 RMB, and participants who completed all three measurements received an additional completion bonus of 20 RMB.

To ensure data quality, two attention check items were embedded in the questionnaire (e.g., “Please select ‘Strongly agree’ for this item”) to identify invalid questionnaires with random responses. A minimum completion time threshold (8 min) was also established, with questionnaires completed below this threshold considered invalid and excluded from analysis. The average completion time for each questionnaire was approximately 15 to 20 min.

### Measures

The core variables in this study include social media feedback (likes and comments), self-esteem, social comparison tendency, depressive symptoms, and anxiety symptoms. All measurement instruments were well-established scales with validated reliability and validity, administered repeatedly across the three time points to meet the requirements for longitudinal analysis.

Social media feedback was measured using an adapted version of the Social Media Interaction Questionnaire developed by Scissors et al. [6]. This scale comprises two dimensions: like feedback (4 items, e.g., “In the past month, how often did your posted content receive likes?”) and comment feedback (4 items, e.g., “In the past month, how often did you receive positive comments?”). Items were rated on a 5-point Likert scale (1 = never, 5 = always), with higher scores indicating higher frequency of receiving positive feedback. Cronbach’s α coefficients for this scale across the three time points were 0.85, 0.87, and 0.86 for the like dimension, and 0.83, 0.85, and 0.84 for the comment dimension.

Self-esteem was measured using the Chinese version of the Rosenberg Self-Esteem Scale [32]. This scale contains 10 items (e.g., “I feel that I have a number of good qualities”), rated on a 4-point Likert scale (1 = strongly disagree, 4 = strongly agree), with five items reverse-scored. Higher total scores indicate higher levels of self-esteem. This scale has been widely validated among Chinese college student populations and demonstrates good psychometric properties. Cronbach’s α coefficients for the three time points in this study were 0.88, 0.89, and 0.90.

Social comparison tendency was assessed using the Chinese revised version of the Iowa-Netherlands Social Comparison Orientation Scale [33]. This scale contains 11 items (e.g., “I frequently compare my accomplishments with those of others”), rated on a 5-point Likert scale (1 = strongly disagree, 5 = strongly agree), with higher scores indicating greater tendency to engage in social comparison. Cronbach’s α coefficients for this scale across the three time points in this study were 0.82, 0.84, and 0.85.

Depressive symptoms were measured using the Patient Health Questionnaire-9 (PHQ-9) [34]. This scale contains 9 items assessing the frequency of depressive symptoms over the past two weeks (e.g., “Little interest or pleasure in doing things”), rated on a 4-point scale (0 = not at all, 3 = nearly every day), with total scores ranging from 0 to 27. Higher scores indicate more severe depressive symptoms. Anxiety symptoms were assessed using the Generalized Anxiety Disorder 7-item scale (GAD-7) [35]. This scale contains 7 items measuring the frequency of anxiety symptoms over the past two weeks (e.g., “Feeling nervous, anxious, or on edge”), using the same 4-point scoring as the PHQ-9, with total scores ranging from 0 to 21. Both scales have been culturally adapted and validated in Chinese populations, demonstrating ideal reliability and validity. Cronbach’s α coefficients for the PHQ-9 across the three time points in this study were 0.89, 0.90, and 0.91, and for the GAD-7 were 0.87, 0.88, and 0.89.

Control variables included participant demographic characteristics (age, gender) and average daily social media usage time. These variables were collected at the baseline time point and included as covariates in subsequent analyses to control for their potential confounding effects.

### Statistical analysis

Data analysis was completed using SPSS 26.0 and Mplus 8.4 statistical software. Prior to formal model testing, missing value analysis and normality testing were conducted. Little’s MCAR test results indicated that data missingness conformed to a missing completely at random pattern (χ² = 142.36, df = 128, *p* = 0.18); therefore, full information maximum likelihood (FIML) estimation was employed to handle missing data, as this method provides unbiased parameter estimates while retaining all available information. Testing of skewness and kurtosis for each variable revealed that all variables had absolute skewness values less than 2 and absolute kurtosis values less than 7, satisfying the basic assumptions of multivariate normal distribution.

To examine the longitudinal relationships between social media feedback and depression/anxiety, as well as the mediating effects of self-esteem and social comparison, this study constructed a cross-lagged panel model (CLPM) [36]. This model enables examination of cross-temporal predictive relationships between variables while controlling for autoregressive effects, thereby providing stronger evidence for temporal precedence and directionality of effects. The model simultaneously incorporated autoregressive paths for each variable between adjacent time points (e.g., depression T1 → depression T2), correlations among variables at the same time point, and cross-lagged paths between different variables (e.g., likes T1 → depression T2). To satisfy the assumption of longitudinal measurement invariance, equality constraints on factor loadings across time points for each scale were tested prior to model estimation.

Model fit was assessed using the following indices: chi-square test (χ²), comparative fit index (CFI), Tucker-Lewis index (TLI), root mean square error of approximation (RMSEA) with 90% confidence interval, and standardized root mean square residual (SRMR). According to structural equation modeling evaluation criteria, CFI and TLI values greater than 0.95, RMSEA less than 0.06, and SRMR less than 0.08 indicate good model fit to the data.

Mediation effects were tested using the bias-corrected bootstrap method, which estimates the sampling distribution and confidence intervals of indirect effects through 5,000 resamples with replacement from the original sample. Compared to the traditional Sobel test, the bootstrap method is more robust to non-normality in the sampling distribution of indirect effects [36]. The criterion for determining the significance of indirect effects was that the 95% bias-corrected confidence interval did not contain zero. Specifically, the mediating effect of self-esteem was tested through the following path: social media feedback (T1) → self-esteem (T2) → depression/anxiety (T3); the mediating effect of social comparison was tested through the path: social media feedback (T1) → social comparison (T2) → depression/anxiety (T3). Effect sizes of indirect effects were expressed as standardized coefficients to facilitate comparison of effect magnitudes across different pathways.

It should be noted that self-esteem and social comparison tendency were modeled as parallel mediators rather than as sequential (serial) mediators in the present study. This analytical decision was based on both methodological and theoretical considerations. Methodologically, both mediator variables were assessed at the same time point (T2), which precludes establishing a temporal sequence between them—a prerequisite for valid serial mediation inference [37]. A rigorous test of serial mediation (e.g., feedback → social comparison → self-esteem → depression) would require at least four measurement occasions with each variable assessed at a distinct time point. Theoretically, sociometer theory and social comparison theory conceptualize self-esteem and social comparison as relatively distinct self-evaluative mechanisms: self-esteem functions as an internalized monitor of social acceptance, whereas social comparison represents an externalized referential process. Although prior research has documented associations between these two constructs, the causal directionality remains debated—some studies suggest that upward social comparison diminishes self-esteem, while others indicate that low self-esteem heightens the propensity for social comparison [38]. Given this theoretical ambiguity regarding their causal ordering, a parallel mediation model represents a more conservative and appropriate specification for the present data.

**Fig. 1:**
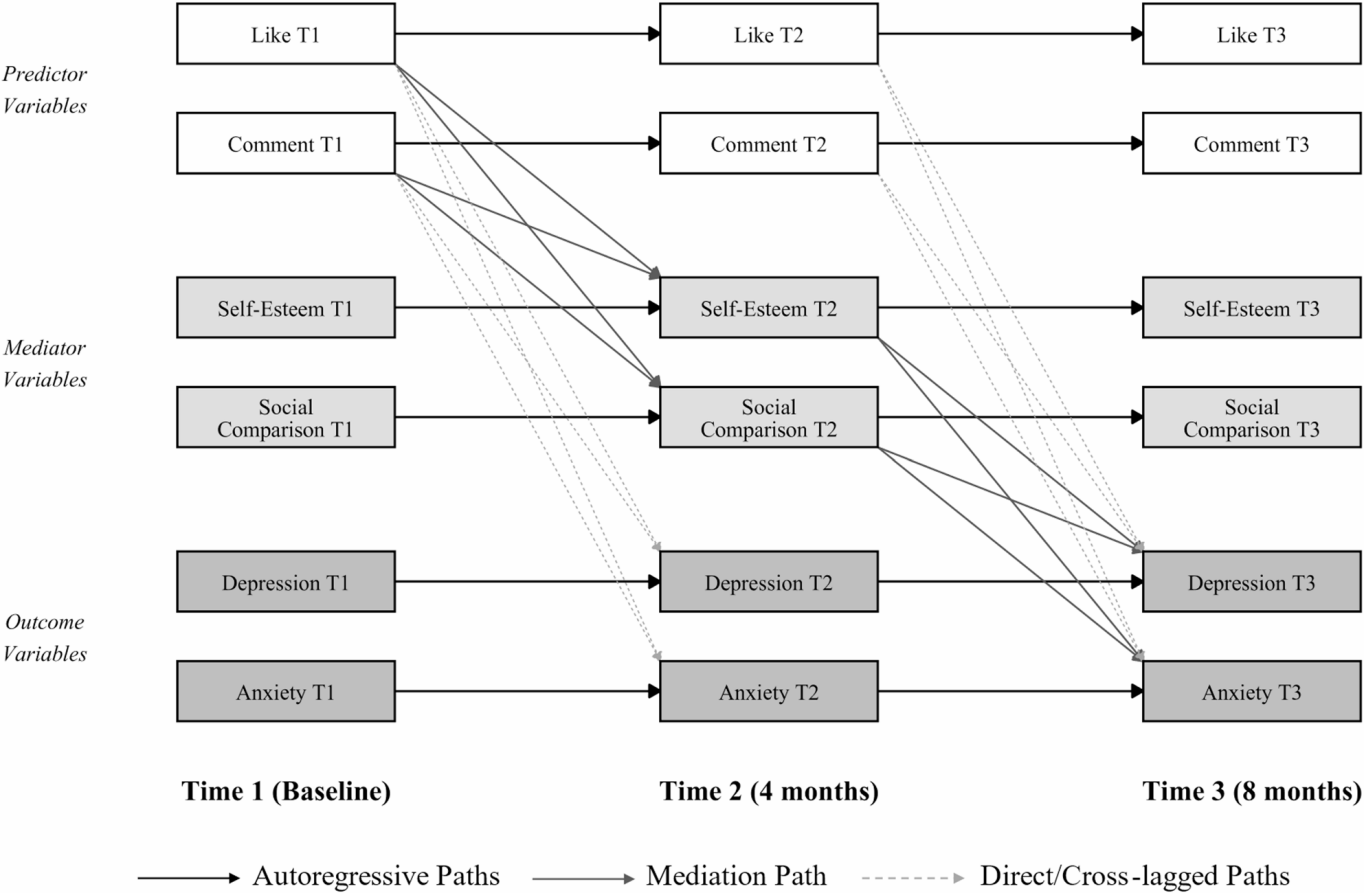
Theoretical hypothesis model

To facilitate intuitive presentation of the analytical strategy and variable relationships in the hypothesized model, Fig. 1 displays the theoretical hypothesis model, including all specified autoregressive paths, cross-lagged paths, and mediation paths.

Figure 1 clearly presents the structure of the longitudinal mediation model tested in this study, including the relationship pathways among social media feedback (likes and comments) as predictor variables, self-esteem and social comparison as mediator variables, and depression and anxiety as outcome variables across the three time points. This model specification provides an analytical framework for the subsequent presentation and interpretation of results.

## Results

### Preliminary analyses

Prior to main hypothesis testing, systematic preliminary analyses were conducted on data quality and sample characteristics. Addressing the common issue of sample attrition in longitudinal research, independent samples t-tests and chi-square tests were used to compare differences in baseline demographic characteristics and core variables between the 398 participants who completed all three measurements and the 88 participants who withdrew. Results showed no statistically significant differences between the two groups in age (t = 1.24, *p* = 0.216), gender distribution (χ² = 0.87, *p* = 0.351), years of education (t = 0.76, *p* = 0.448), or average daily social media usage time (t = 0.93, *p* = 0.354). Regarding core research variables, the two groups showed no significant differences in baseline like feedback frequency (t = 1.08, *p* = 0.281), comment feedback frequency (t = 0.89, *p* = 0.374), self-esteem levels (t = 1.32, *p* = 0.187), social comparison tendency (t = 0.95, *p* = 0.343), depressive symptoms (t = 1.41, *p* = 0.159), and anxiety symptoms (t = 1.18, *p* = 0.238), indicating that sample attrition exhibited random characteristics and would not produce systematic bias in subsequent analysis results.

Normality testing was conducted for all core variables. Results showed that absolute skewness values for all variables across the three time points ranged from 0.31 to 0.89, and absolute kurtosis values ranged from 0.24 to 1.12, all within acceptable ranges (skewness < 2, kurtosis < 7), satisfying the basic assumptions for subsequent parametric tests. Descriptive statistics for each variable across the three time points and bivariate Pearson correlation coefficients among variables are presented in Table 2.

**Table 2:** Descriptive statistics and correlations among study variables

Variable	M	SD	1	2	3	4	5	6	7	8
1. Like T1	2.89	0.94	—							
2. Like T2	2.85	0.91	0.62**	—						
3. Like T3	2.82	0.93	0.54**	0.58**	—					
4. Comment T1	2.64	0.88	0.52**	0.46**	0.43**	—				
5. Comment T2	2.59	0.85	0.48**	0.55**	0.47**	0.59**	—			
6. Comment T3	2.56	0.87	0.44**	0.49**	0.58**	0.54**	0.54**	—		
7. Self-Esteem T1	28.46	5.12	0.34**	0.29**	0.26**	0.30**	0.26**	0.24**	—	
8. Self-Esteem T2	28.21	5.08	0.31**	0.36**	0.28**	0.27**	0.32**	0.26**	0.65**	—
9. Self-Esteem T3	27.94	5.23	0.28**	0.30**	0.35**	0.25**	0.28**	0.33**	0.59**	0.61**
10. Social Comparison T1	35.72	7.84	−0.28**	−0.24**	−0.22**	−0.25**	−0.22**	−0.20**	−0.31**	−0.27**
11. Social Comparison T2	36.15	7.92	−0.25**	−0.30**	−0.24**	−0.23**	−0.27**	−0.22**	−0.28**	−0.33**
12. Social Comparison T3	36.58	8.03	−0.23**	−0.26**	−0.31**	−0.21**	−0.24**	−0.28**	−0.26**	−0.29**
13. Depression T1	6.72	4.85	−0.38**	−0.33**	−0.31**	−0.35**	−0.30**	−0.28**	−0.51**	−0.45**
14. Depression T2	6.89	4.92	−0.35**	−0.36**	−0.32**	−0.32**	−0.33**	−0.29**	−0.47**	−0.49**
15. Depression T3	7.14	5.03	−0.32**	−0.33**	−0.35**	−0.29**	−0.30**	−0.32**	−0.44**	−0.46**
16. Anxiety T1	5.38	4.21	−0.34**	−0.29**	−0.27**	−0.31**	−0.27**	−0.24**	−0.44**	−0.39**
17. Anxiety T2	5.52	4.34	−0.30**	−0.32**	−0.28**	−0.28**	−0.30**	−0.26**	−0.40**	−0.43**
18. Anxiety T3	5.71	4.46	−0.28**	−0.29**	−0.31**	−0.25**	−0.27**	−0.29**	−0.37**	−0.40**

Variable	9	10	11	12	13	14	15	16	17	18
1. Like T1										
2. Like T2										
3. Like T3										
4. Comment T1										
5. Comment T2										
6. Comment T3										
7. Self-Esteem T1										
8. Self-Esteem T2										
9. Self-Esteem T3	—									
10. Social Comparison T1	−0.25**	—								
11. Social Comparison T2	−0.28**	0.60**	—							
12. Social Comparison T3	−0.34**	0.55**	0.56**	—						
13. Depression T1	−0.42**	0.37**	0.32**	0.30**	—					
14. Depression T2	−0.44**	0.34**	0.36**	0.32**	0.57**	—				
15. Depression T3	−0.48**	0.31**	0.33**	0.35**	0.52**	0.52**	—			
16. Anxiety T1	−0.36**	0.33**	0.28**	0.26**	0.68**	0.54**	0.49**	—		
17. Anxiety T2	−0.38**	0.30**	0.32**	0.28**	0.55**	0.67**	0.51**	0.54**	—	
18. Anxiety T3	−0.42**	0.27**	0.29**	0.31**	0.50**	0.52**	0.66**	0.49**	0.49**	—

*N* = 398. *T1* = Time 1 (baseline); *T2* = Time 2 (4-month follow-up); *T3* = Time 3 (8-month follow-up). *Like* = Like feedback; *Comment* = Comment feedback. **p < 0.01

The descriptive statistics presented in Table 2 showed that the mean values for like feedback across the three time points were 2.89 (SD = 0.94), 2.85 (SD = 0.91), and 2.82 (SD = 0.93), respectively. The mean values for comment feedback were 2.64 (SD = 0.88), 2.59 (SD = 0.85), and 2.56 (SD = 0.87), indicating that the overall frequency of receiving positive social media feedback among participants was at a moderate to low level and remained relatively stable throughout the eight-month tracking period, showing no significant temporal trends. Self-esteem scale scores at the three time points had mean values of 28.46 (SD = 5.12), 28.21 (SD = 5.08), and 27.94 (SD = 5.23), indicating moderate to high levels. Social comparison tendency mean values were 35.72 (SD = 7.84), 36.15 (SD = 7.92), and 36.58 (SD = 8.03), showing a slight upward trend. Depressive symptoms (PHQ-9) mean values at the three time points were 6.72 (SD = 4.85), 6.89 (SD = 4.92), and 7.14 (SD = 5.03), and anxiety symptoms (GAD-7) mean values were 5.38 (SD = 4.21), 5.52 (SD = 4.34), and 5.71 (SD = 4.46), overall within the mild symptom range but showing a slight upward trend over time.

Correlation analysis results presented in Table 2 showed that the correlation patterns among variables were generally consistent with theoretical expectations. Regarding associations between social media feedback and mental health outcome variables, like feedback was significantly negatively correlated with depressive symptoms both concurrently and cross-temporally, with correlation coefficients ranging from *r* = − 0.31 to − 0.38 (all *p* < 0.01); the negative correlations with anxiety symptoms were slightly weaker, ranging from *r* = − 0.27 to − 0.34 (all *p* < 0.01). Comment feedback correlations with depressive symptoms ranged from *r* = − 0.28 to − 0.35 (all *p* < 0.01), and with anxiety symptoms from *r* = − 0.24 to − 0.31 (all *p* < 0.01), showing a similar pattern to like feedback but with slightly smaller effect sizes. Regarding associations between mediator and outcome variables, self-esteem showed moderate negative correlations with depressive symptoms (*r* = − 0.42 to − 0.51, *p* < 0.001), and somewhat weaker negative correlations with anxiety symptoms (*r* = − 0.36 to − 0.44, *p* < 0.001). Social comparison tendency was significantly positively correlated with both depression (*r* = 0.29 to 0.37, *p* < 0.01) and anxiety (*r* = 0.25 to 0.33, *p* < 0.01) symptoms. Autocorrelation coefficients for the same variable across different time points ranged from 0.54 to 0.68, indicating moderate cross-temporal stability while also exhibiting sufficient within-individual variability to support testing of cross-lagged effects. Like feedback and comment feedback were moderately positively correlated (*r* = 0.52 to 0.58, *p* < 0.001), indicating that both types of feedback share common variance while maintaining relative independence, supporting their analysis as two independent predictor variables in the model.

### Measurement model

Prior to constructing the cross-lagged panel model, systematic tests of factor structure and measurement invariance were conducted for each scale. Confirmatory factor analyses were performed separately for the measurement models of the five core variables (like feedback, comment feedback, self-esteem, social comparison, depression, and anxiety) at each time point. Results showed that all scales exhibited good factor structures across the three time points, with fit indices reaching acceptable standards (all CFI > 0.92, all RMSEA < 0.07) and factor loadings ranging from 0.58 to 0.84, all reaching statistical significance (*p* < 0.001).

Longitudinal measurement invariance was tested using a sequential constraint strategy. The configural invariance model yielded the following fit indices: χ²(486) = 892.45, CFI = 0.958, TLI = 0.951, RMSEA = 0.046. Building on this, equality constraints on factor loadings across time points were imposed for each variable to test weak (metric) measurement invariance. Results indicated that the weak measurement invariance model did not show significant deterioration in fit compared to the configural invariance model (Δχ²(24) = 31.56, *p* = 0.138; ΔCFI = 0.003; ΔRMSEA = 0.002), indicating that the measurement instruments possessed equivalent measurement properties across the three time points. Further testing of strong (scalar) measurement invariance through imposing equality constraints on item intercepts similarly supported the measurement invariance assumption (ΔCFI = 0.005; ΔRMSEA = 0.003), satisfying the prerequisite assumptions for longitudinal comparison and cross-lagged analysis.

### Cross-lagged panel model

After confirming measurement invariance, a cross-lagged panel model was constructed to test the longitudinal relationships between social media feedback and depression/anxiety [36]. The model simultaneously incorporated autoregressive paths for each variable between adjacent time points, covariance relationships among variables at the same time point, and cross-lagged paths between different variables, with age, gender, and average daily social media usage time controlled as covariates. The overall model fit indices indicated good fit between the model and the data: χ² (142) = 246.83, *p* < 0.001; CFI = 0.962; TLI = 0.954; RMSEA = 0.043, 90% CI [0.034, 0.052]; SRMR = 0.038. Although the chi-square test reached significance, considering the sensitivity of this index to sample size and the comprehensive assessment of other fit indices, the model fit can be considered ideal.

**Fig. 2:**
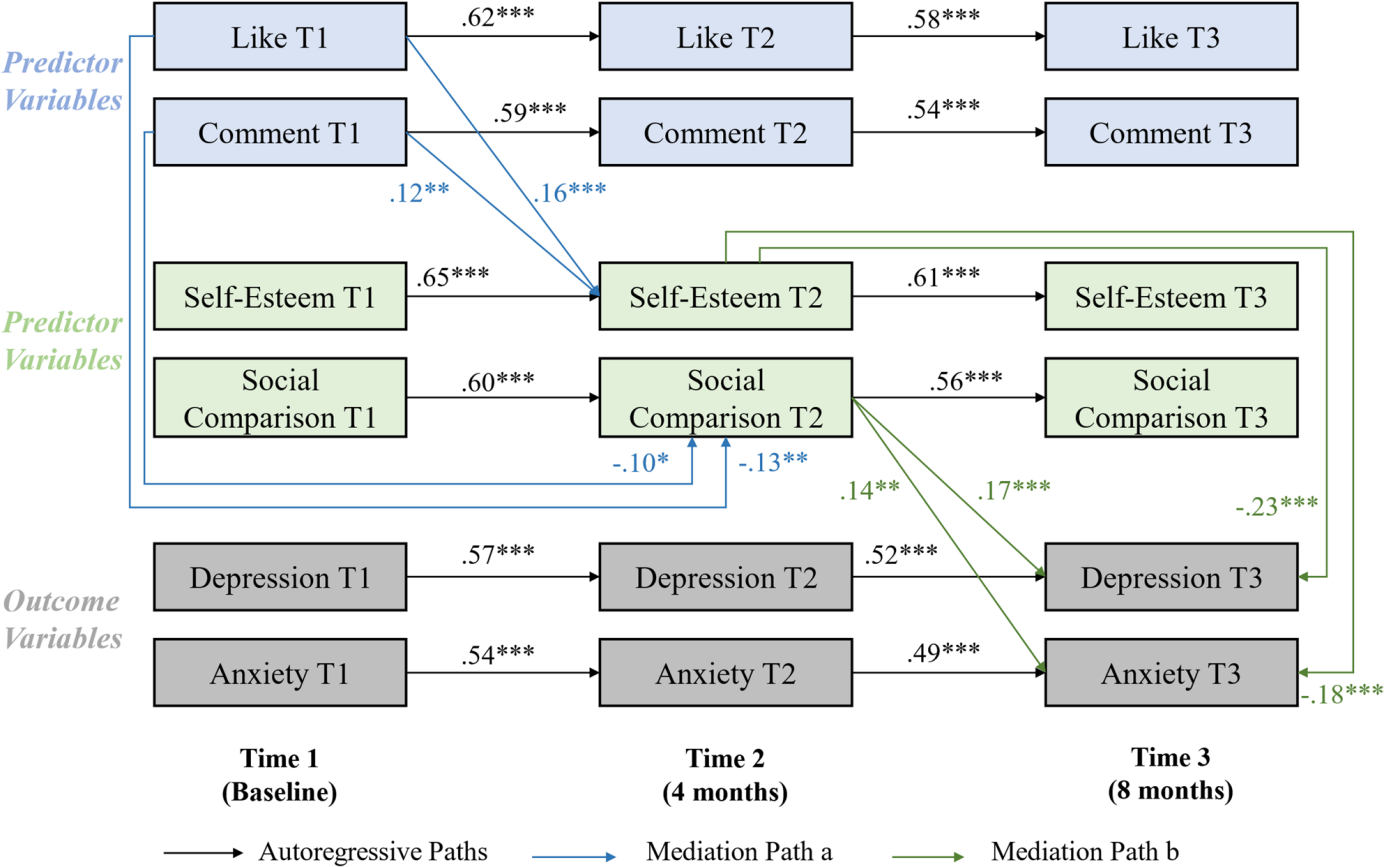
Cross-lagged panel model results

All autoregressive paths for all variables in the model reached statistical significance (*p* < 0.001), confirming moderate stability of each variable during the tracking period. The autoregressive coefficients for like feedback were β = 0.62 from T1 to T2 and β = 0.58 from T2 to T3; corresponding coefficients for comment feedback were β = 0.59 and β = 0.54, respectively. For mediator variables, autoregressive coefficients for self-esteem were β = 0.65 (T1→T2) and β = 0.61 (T2→T3), and for social comparison tendency were β = 0.60 and β = 0.56. Outcome variable stability was also at moderate levels, with autoregressive coefficients for depressive symptoms at β = 0.57 (T1→T2) and β = 0.52 (T2→T3), and for anxiety symptoms at β = 0.54 and β = 0.49. The presence of these autoregressive effects indicates that testing cross-lagged effects while controlling for variables’ own cross-temporal stability is methodologically necessary and appropriate. Complete path coefficients for the cross-lagged panel model are presented in Table 3, and a diagram of the standardized path coefficients is shown in Fig. 2.

**Table 3:** Cross-lagged path coefficients

Path	β	SE	*p*
Autoregressive Paths			
Like T1 → Like T2	0.62	0.03	< 0.001
Like T2 → Like T3	0.58	0.04	< 0.001
Comment T1 → Comment T2	0.59	0.03	< 0.001
Comment T2 → Comment T3	0.54	0.04	< 0.001
Self-Esteem T1 → Self-Esteem T2	0.65	0.03	< 0.001
Self-Esteem T2 → Self-Esteem T3	0.61	0.03	< 0.001
Social Comparison T1 → Social Comparison T2	0.60	0.03	< 0.001
Social Comparison T2 → Social Comparison T3	0.56	0.04	< 0.001
Depression T1 → Depression T2	0.57	0.04	< 0.001
Depression T2 → Depression T3	0.52	0.04	< 0.001
Anxiety T1 → Anxiety T2	0.54	0.04	< 0.001
Anxiety T2 → Anxiety T3	0.49	0.04	< 0.001
Cross-Lagged Paths: Feedback → Mental Health			
Like T1 → Depression T2	−0.14	0.04	< 0.01
Like T1 → Anxiety T2	−0.11	0.04	< 0.05
Like T2 → Depression T3	−0.12	0.04	< 0.01
Like T2 → Anxiety T3	−0.09	0.04	< 0.05
Comment T1 → Depression T2	−0.11	0.04	< 0.05
Comment T1 → Anxiety T2	−0.09	0.04	< 0.05
Comment T2 → Depression T3	−0.09	0.04	< 0.05
Comment T2 → Anxiety T3	−0.07	0.04	0.062
Cross-Lagged Paths: Feedback → Mediators			
Like T1 → Self-Esteem T2	0.16	0.04	< 0.001
Like T1 → Social Comparison T2	−0.13	0.04	< 0.01
Comment T1 → Self-Esteem T2	0.12	0.04	< 0.01
Comment T1 → Social Comparison T2	−0.10	0.04	< 0.05
Cross-Lagged Paths: Mediators → Mental Health			
Self-Esteem T2 → Depression T3	−0.23	0.04	< 0.001
Self-Esteem T2 → Anxiety T3	−0.18	0.04	< 0.001
Social Comparison T2 → Depression T3	0.17	0.04	< 0.001
Social Comparison T2 → Anxiety T3	0.14	0.04	< 0.01
Reverse Paths: Mental Health → Feedback			
Depression T1 → Like T2	−0.04	0.04	0.312
Depression T1 → Comment T2	−0.03	0.04	0.426
Anxiety T1 → Like T2	−0.02	0.04	0.548
Anxiety T1 → Comment T2	−0.02	0.04	0.612

*N* = 398. *T1* = Time 1 (baseline); *T2* = Time 2 (4-month follow-up); *T3* = Time 3 (8-month follow-up). All coefficients are standardized

Model fit: χ^2^(142) = 246.83,*p* < 0.001; CFI = 0.962; TLI = 0.954; RMSEA = 0.043, 90% CI [0.034, 0.052]; SRMR = 0.038. Age, gender, and daily social media usage time were controlled as covariates

The cross-lagged path results presented in Table 3; Fig. 2 provided empirical support for the research hypotheses. Regarding the predictive effects of social media feedback on mental health outcomes, consistent with Hypothesis H1, T1 like feedback significantly negatively predicted T2 depressive symptoms (β = −0.14, SE = 0.04, *p* < 0.01) and anxiety symptoms (β = −0.11, SE = 0.04, *p* < 0.05), indicating that individuals who received lower frequencies of like feedback exhibited higher levels of depressive and anxiety symptoms four months later. The corresponding paths from T2 to T3 were also significant (depression: β = −0.12, SE = 0.04, *p* < 0.01; anxiety: β = −0.09, SE = 0.04, *p* < 0.05), confirming the replicability of this longitudinal effect across different time intervals. Consistent with Hypothesis H2, the cross-temporal predictive effects of comment feedback on depressive and anxiety symptoms also reached statistical significance (T1→T2 depression: β = −0.11, SE = 0.04, *p* < 0.05; T1→T2 anxiety: β = −0.09, SE = 0.04, *p* < 0.05), with effect sizes slightly smaller than those for like feedback. The predictive effects of comment feedback in the T2→T3 interval showed a similar pattern (depression: β = −0.09, *p* < 0.05; anxiety: β = −0.07, *p* = 0.062), with the predictive effect on anxiety approaching but not reaching the conventional significance threshold.

Regarding the predictive effects of social media feedback on mediator variables, T1 like feedback significantly positively predicted T2 self-esteem (β = 0.16, SE = 0.04, *p* < 0.001) and significantly negatively predicted social comparison tendency (β = −0.13, SE = 0.04, *p* < 0.01). The predictive effects of comment feedback on self-esteem (β = 0.12, *p* < 0.01) and social comparison (β = −0.10, *p* < 0.05) were also significant but with smaller effect sizes. Regarding the predictive effects of mediator variables on outcome variables, T2 self-esteem significantly negatively predicted T3 depressive (β = −0.23, SE = 0.04, *p* < 0.001) and anxiety (β = −0.18, SE = 0.04, *p* < 0.001) symptoms. Social comparison tendency significantly positively predicted depressive (β = 0.17, SE = 0.04, *p* < 0.001) and anxiety (β = 0.14, SE = 0.04, *p* < 0.01) symptoms.

Reverse path testing results showed that T1 depressive symptoms did not significantly predict T2 like feedback (β = −0.04, *p* = 0.312) or comment feedback (β = −0.03, *p* = 0.426). The reverse predictive effects of anxiety symptoms on social media feedback were also non-significant (on likes: β = −0.02, *p* = 0.548; on comments: β = −0.02, *p* = 0.612). Reverse paths in the T2 to T3 interval showed consistent non-significant patterns (all *p* > 0.10). These results indicate that the influence of social media feedback on mental health is directionally specific—that is, feedback influences mental health rather than the reverse—providing preliminary evidence for the temporal directionality of the relationships between variables.

### Mediation effects

After verifying the direct longitudinal effects of social media feedback on depression/anxiety as well as the predictive effects of feedback on mediators and mediators on outcomes, the longitudinal mediating effects of self-esteem and social comparison were formally tested using the bias-corrected bootstrap method (5,000 resamples) [31]. Mediation analysis was based on the following longitudinal path: social media feedback (T1) → mediator variable (T2) → mental health outcome (T3). This temporal design ensures the sequential ordering of predictor, mediator, and outcome variables in time, enhancing the causal inference strength of mediation effect interpretation. Complete results of mediation effect analysis are presented in Table 4, including indirect effect values, standard errors, and 95% bias-corrected confidence intervals for each mediation pathway.

**Table 4:** Mediation effect test results

Path	Effect	SE	95% CI	% of Total
Like Feedback → Depression				
Total effect	−0.140	0.038	[− 0.214, − 0.066]	—
Direct effect	−0.049	0.024	[− 0.096, − 0.002]*	35.0%
Indirect effect via Self-Esteem	−0.052	0.016	[− 0.086, − 0.024]*	37.1%
Indirect effect via Social Comparison	−0.039	0.013	[− 0.067, − 0.016]*	27.9%
Total indirect effect	−0.091	0.020	[− 0.132, − 0.054]*	65.0%
Like Feedback → Anxiety				
Total effect	−0.110	0.036	[− 0.180, − 0.040]	—
Direct effect	−0.036	0.020	[− 0.075, 0.003]	32.7%
Indirect effect via Self-Esteem	−0.041	0.014	[− 0.071, − 0.017]*	37.3%
Indirect effect via Social Comparison	−0.033	0.012	[− 0.061, − 0.011]*	30.0%
Total indirect effect	−0.074	0.018	[− 0.111, − 0.041]*	67.3%
Comment Feedback → Depression				
Total effect	−0.110	0.037	[− 0.182, − 0.038]	—
Direct effect	−0.041	0.022	[− 0.084, 0.002]	37.3%
Indirect effect via Self-Esteem	−0.038	0.013	[− 0.069, − 0.014]*	34.5%
Indirect effect via Social Comparison	−0.028	0.011	[− 0.052, − 0.009]*	25.5%
Total indirect effect	−0.066	0.017	[− 0.101, − 0.035]*	60.0%
Comment Feedback → Anxiety				
Total effect	−0.090	0.035	[− 0.158, − 0.022]	—
Direct effect	−0.032	0.020	[− 0.071, 0.007]	35.6%
Indirect effect via Self-Esteem	−0.031	0.012	[− 0.058, − 0.010]*	34.4%
Indirect effect via Social Comparison	−0.023	0.010	[− 0.045, − 0.006]*	25.6%
Total indirect effect	−0.054	0.015	[− 0.086, − 0.027]*	60.0%

*N* = 398. *CI* = confidence interval. All indirect effects were tested using bias-corrected bootstrap method with 5,000 resamples. The mediation path is: Social media feedback (T1) → Mediator (T2) → Mental health outcome (T3). Percentage of total effect was calculated as (indirect effect/total effect) × 100%. * 95% bias-corrected confidence interval does not contain zero, indicating significant effect

The results in Table 4 provided supporting evidence for Hypotheses H3 and H4. Testing of the mediating effect of self-esteem showed that the indirect effect of like feedback on depressive symptoms through self-esteem was significant (indirect effect = − 0.052, SE = 0.016, 95% CI [− 0.086, − 0.024]). This confidence interval does not contain zero, supporting the mediating role of self-esteem. This pathway indicates that lower frequency of like feedback predicts lower self-esteem levels (path a: β = 0.16), and lower self-esteem in turn predicts higher depressive symptoms (path b: β = −0.23). The indirect effect of like feedback on anxiety symptoms through self-esteem was also significant (indirect effect = − 0.041, SE = 0.014, 95% CI [− 0.071, − 0.017]). The indirect effects of comment feedback on depression (indirect effect = − 0.038, SE = 0.013, 95% CI [− 0.069, − 0.014]) and anxiety (indirect effect = − 0.031, SE = 0.012, 95% CI [− 0.058, − 0.010]) through self-esteem also reached significance, though with relatively smaller effect sizes, consistent with the finding that comment feedback’s predictive effect on self-esteem (β = 0.12) was weaker than that of like feedback (β = 0.16).

Testing results for social comparison as a mediator also supported the research hypotheses. The indirect effect of like feedback on depressive symptoms through social comparison tendency was significant (indirect effect = − 0.039, SE = 0.013, 95% CI [− 0.067, − 0.016]), indicating that lower frequency of like feedback was associated with stronger social comparison tendency (path a: β = −0.13), which in turn predicted higher depression levels (path b: β = 0.17). The indirect effect of like feedback on anxiety symptoms through social comparison was also significant (indirect effect = − 0.033, SE = 0.012, 95% CI [− 0.061, − 0.011]). The indirect effects of comment feedback on depression (indirect effect = − 0.028, 95% CI [− 0.052, − 0.009]) and anxiety (indirect effect = − 0.023, 95% CI [− 0.045, − 0.006]) through social comparison showed similar patterns, with 95% confidence intervals not containing zero.

To further quantify the relative importance of each mediation pathway, a decomposition analysis of the proportion of mediation effects to total effects was conducted. Taking the effect of like feedback on depressive symptoms as an example, the total effect (c = − 0.140) can be decomposed into direct effect (c’ = −0.049) and two indirect effect pathways: indirect effect through self-esteem (a₁ × b₁ = −0.052) and indirect effect through social comparison (a₂ × b₂ = −0.039). Based on the formula: Proportion of mediation effect = (a × b/c) × 100%, the mediating effect of self-esteem accounted for 37.1% of the total effect in the relationship between like feedback and depressive symptoms (− 0.052/−0.140 = 0.371), and the mediating effect of social comparison accounted for 27.9% (− 0.039/−0.140 = 0.279), with the two mediators together explaining 65.0% of the total effect. In the relationship between like feedback and anxiety symptoms, the mediating effects of self-esteem and social comparison accounted for 37.3% and 30.0% of the total effect, respectively, together explaining 67.3%. The decomposition of mediation effects for the impact of comment feedback on depression and anxiety showed similar patterns, with the proportion of self-esteem mediation (34.5% to 36.5%) slightly higher than that of social comparison (25.5% to 28.2%). These results indicate that the effects of social media feedback on mental health are largely transmitted through these two self-evaluation-related psychological mechanisms: self-esteem and social comparison.

After including mediator variables, the direct effect of like feedback on depressive symptoms was attenuated but remained significant (β = −0.049, SE = 0.024, *p* = 0.041, 95% CI [− 0.096, − 0.002]), while the direct effect of like feedback on anxiety symptoms was marginally significant (β = −0.036, *p* = 0.068). The direct effects of comment feedback on depression (β = −0.041, *p* = 0.058) and anxiety (β = −0.032, *p* = 0.112) after controlling for mediators did not reach significance. These results suggest that self-esteem and social comparison play partial mediating roles in the relationship between like feedback and depressive symptoms, while they may play complete mediating roles in the relationship between comment feedback and mental health outcomes.

### Supplementary analyses

To test the robustness of research findings and explore potential moderation effects, several supplementary analyses were conducted. Multi-group analysis was used to test whether gender moderated the longitudinal effects of social media feedback on mental health [11]. By comparing the fit differences between constrained models (cross-lagged paths constrained to be equal across gender groups) and unconstrained models, results showed no significant difference in fit indices between the two models (Δχ² (8) = 11.24, *p* = 0.188; ΔCFI = 0.003), indicating that the longitudinal effects of social media feedback on depression and anxiety, as well as the mediation effect patterns, showed cross-gender consistency among male and female participants, with no significant gender moderation effects.

Further analysis incorporated average daily social media usage time as a moderator to test whether social media usage intensity affected the magnitude of feedback effects. Using the median (3 h/day) as a cutoff, the sample was divided into high-use (*n* = 187) and low-use (*n* = 211) groups. Multi-group comparison results showed no significant differences in cross-lagged path coefficients between the two groups (Δχ² (8) = 9.86, *p* = 0.275), suggesting that the effects of social media feedback on mental health remained relatively stable across users with different usage intensities.

As a sensitivity analysis, a random intercepts cross-lagged panel model (RI-CLPM) was used to supplement the verification of results [39]. This model separates between-person differences from within-person changes by introducing random intercepts, enabling more precise estimation of cross-temporal dynamic relationships between variables. RI-CLPM results showed that the direction and significance patterns of core cross-lagged paths were consistent with the traditional CLPM, with the within-person predictive effect of like feedback on depressive symptoms remaining significant (β = −0.11, *p* < 0.05), further supporting the robustness of research conclusions.

## Discussion

The present study employed a three-wave longitudinal tracking design to systematically examine the longitudinal effects of social media likes and comments feedback on depressive and anxiety symptoms among young adult users, and tested the mediating roles of self-esteem and social comparison in this relationship. Results demonstrated that lower frequency of positive social media feedback significantly predicted higher levels of depressive and anxiety symptoms at subsequent time points, with self-esteem and social comparison tendency together explaining 65.0% of this effect, revealing the core psychological mechanisms through which social media feedback influences the mental health of young adults.

### Longitudinal effects of social media feedback on mental health

This study found that lower frequency of like feedback significantly predicted subsequent higher levels of depressive (β = −0.14) and anxiety (β = −0.11) symptoms. This result is consistent with association patterns revealed by prior cross-sectional studies [3, 4], but the longitudinal design further established the temporal directionality between variables. Nesi and Prinstein’s research showed that adolescents who received less positive feedback exhibited higher levels of psychological distress [13], and the present study provides stronger longitudinal evidence supporting the temporal precedence of feedback in predicting subsequent mental health outcomes.

The effect sizes observed in the present study warrant contextualization within the broader literature. The cross-lagged effect of like feedback on depressive symptoms (β = −0.14) and anxiety symptoms (β = −0.11) fall within the range of small to moderate effects according to conventional benchmarks in social and behavioral sciences [40]. These magnitudes are comparable to or slightly larger than those reported in meta-analyses of the association between social media use and mental health. For instance, Huang (2017) [3] reported a weighted mean correlation of *r* = 0.13 between time spent on social media and poor psychological well-being, and Shannon et al. (2022) [5] found pooled effect sizes in the range of *r* = 0.10 to 0.19 for associations between problematic social media use and internalizing symptoms in young populations. Notably, the effect sizes in the present study were obtained from cross-lagged models that controlled for prior levels of the outcome variable—a more conservative approach than the concurrent correlational designs used in most meta-analyzed studies—suggesting that the true longitudinal effects of social media feedback may be meaningful despite their modest absolute magnitude. As Funder and Ozer (2019) have argued [41], small effects in longitudinal research can accumulate over time and carry substantial real-world significance when they operate on a large population over repeated exposures, which is precisely the case with daily social media feedback experienced by millions of young adults.

Notably, the reverse cross-lagged paths from depression and anxiety symptoms to subsequent social media feedback were consistently non-significant across both time intervals. This finding warrants careful interpretation rather than a simple conclusion that no reverse influence exists. Several considerations are relevant here.

First, the non-significant reverse paths may reflect a genuine asymmetry in the direct relationship between these variables. Unlike general social media usage behaviors—which individuals can actively increase or decrease in response to their emotional states—the amount of feedback one receives is largely determined by others’ behaviors and is therefore less directly controllable by the individual. This distinction may explain why feedback predicts subsequent emotional outcomes, but emotional states do not directly predict subsequent feedback received.

Second, and importantly, the absence of significant direct reverse paths does not preclude the existence of indirect reverse effects. As the reviewer insightfully noted, depression and anxiety may influence received feedback through intermediary behavioral changes that were not measured in the present study. Specifically, depressed or anxious individuals may reduce their posting frequency due to social withdrawal, diminished motivation, or reduced interest in social interaction—all of which are core features of depression [42]. With fewer posts published, there are naturally fewer opportunities to receive likes and comments. Additionally, when individuals experiencing psychological distress do post content, their posts may carry a more negative emotional tone, which has been shown to elicit fewer positive reactions and less engagement from others in their social network [43]. Through either of these pathways—reduced posting quantity or more negative posting content—depression and anxiety could indirectly lead to decreased positive feedback over time. However, because the present model did not include posting behavior (frequency or content valence) as a variable, these indirect reverse pathways could not be captured by the direct cross-lagged paths tested.

Third, the four-month interval between measurement occasions may not be optimally suited to detect reverse effects. It is possible that the impact of emotional states on social media behavior operates on a shorter timescale (days to weeks rather than months), and such effects may have dissipated or been obscured within the longer measurement interval used in this study [44].

Taken together, while the present findings provide evidence for the temporal precedence of social media feedback in predicting subsequent mental health outcomes, we caution against concluding that reverse influence is entirely absent. Future research should incorporate measures of posting behavior—including posting frequency, content valence, and active versus passive social media use patterns—as potential mediating variables in the reverse pathway to provide a more comprehensive understanding of the bidirectional dynamics between social media engagement and mental health. The predictive effects of like feedback were slightly stronger than those of comment feedback, consistent with the conclusions of Scissors et al. [6], who noted that likes, as a low-cost, high-frequency, and quantifiable signal of social approval, hold unique psychological significance when users evaluate their own social worth.

### The mediating roles of self-esteem and social comparison

The mediating effect of self-esteem in the relationship between social media feedback and mental health accounted for 37.1% of the total effect, a finding highly consistent with sociometer theory proposed by Leary and colleagues [22]. This theory posits that self-esteem serves as an internal indicator through which individuals monitor their degree of social acceptance, dynamically adjusting in response to signals of acceptance or rejection from the social environment, with likes and comments being viewable as digitized signals of social acceptance [23]. The present findings are also consistent with the social media self-esteem susceptibility model proposed by Valkenburg et al. [25, 26], which emphasizes that social media feedback has immediate and significant effects on adolescent self-esteem, and that fluctuations in self-esteem are closely related to subsequent emotional problems.

Social comparison, as another mediator variable, explained 27.9% of the total effect, providing empirical support for the application of Festinger’s social comparison theory in digitized social contexts [28]. Social media platforms, through visually presenting like and comment counts, create highly salient social comparison cues for users [29, 30]. When individuals find that they receive fewer feedbacks than others, such upward social comparison may trigger feelings of relative deprivation, thereby increasing the risk of psychological symptoms [17]. Moreton and Greenfeld’s qualitative study of British university students revealed a similar process, with participants commonly associating like counts with their own popularity [15]. The two mediator variables together explained most of the total effect, but the direct effect remained partially significant after including mediators, suggesting that other unmeasured mechanisms may exist, such as sleep quality or fear of missing out, which warrant future exploration. It is worth noting that the two mediating mechanisms, while modeled as parallel pathways, are not entirely independent of each other. The moderate negative correlations between self-esteem and social comparison tendency observed across time points (*r* = − 0.28 to − 0.34) suggest potential interplay between these constructs. Theoretically, it is plausible that social media feedback initiates a cascading process in which heightened social comparison erodes self-esteem, or conversely, that diminished self-esteem amplifies the tendency to engage in social comparison. However, because both mediators were measured concurrently at T2, the present study cannot empirically disentangle these possibilities. The finding that self-esteem accounted for a somewhat larger proportion of the total effect (37.1%) than social comparison (27.9%) may tentatively suggest that the internalized self-evaluative mechanism plays a relatively more central role, but this interpretation should be treated with caution given the parallel model specification. Future research employing four or more measurement waves—with self-esteem and social comparison assessed at separate time points—would allow for rigorous testing of serial mediation pathways and provide a more complete picture of the temporal dynamics between these two self-evaluative processes.

### Theoretical implications

The present findings carry several implications for the theoretical understanding of how social media influences mental health among young adults.

First, the results extend sociometer theory into the digital social context by demonstrating that social media likes and comments function as a form of “digital sociometer.” In its original formulation, sociometer theory conceptualized self-esteem as an internal monitor that tracks cues of social acceptance and rejection in face-to-face interpersonal interactions. The present study provides longitudinal evidence that this monitoring function extends to the digital domain, where likes and comments serve as quantified, publicly visible signals of social acceptance. This finding suggests that the fundamental psychological mechanism described by sociometer theory—the link between perceived social acceptance and self-esteem—operates similarly across offline and online social environments. However, the digital context also introduces unique features that may amplify this mechanism. Unlike face-to-face social cues, which are often ambiguous and transient, social media feedback is numerically precise, permanently recorded, and publicly comparable, potentially making the digital sociometer both more sensitive and more consequential for self-esteem regulation than its offline counterpart.

Second, the present study contributes to social comparison theory by identifying the specific structural features of social media that facilitate comparison processes. Our finding that lower feedback frequency predicted increased social comparison tendency suggests that the quantified nature of social media feedback—particularly the numerical display of like counts—creates a ready-made metric for interpersonal comparison that may not have an equivalent in offline social interactions. This aligns with and extends the theoretical proposition that social media platforms, through their design architecture, create what might be termed “comparison-rich environments” in which opportunities for self-evaluative comparison are both more frequent and more precisely quantifiable than in traditional social settings.

Third, and perhaps most notably, the relative magnitudes of the two mediating pathways offer theoretical insight into the psychological primacy of different self-evaluative mechanisms. Self-esteem consistently accounted for a larger proportion of the total effect (34.5%–37.3%) than social comparison (25.5%–30.0%) across all tested pathways. This pattern suggests that in the context of social media feedback, the internalized self-evaluative mechanism (self-esteem as a sociometer) may play a more central role than the externalized referential mechanism (social comparison). One possible interpretation is that social media feedback is first processed through the sociometer system as a direct signal of one’s social acceptance, and that social comparison represents a secondary, more cognitively effortful process that requires additional contextual information (i.e., knowledge of how much feedback others have received). This hierarchical processing interpretation, while speculative, offers a testable proposition for future research: if self-esteem responses to feedback are indeed more automatic and primary, they should manifest more rapidly and consistently than comparison-driven effects, a hypothesis that could be examined using experience sampling methods with finer temporal resolution.

Fourth, the integration of sociometer theory and social comparison theory within a unified empirical framework reveals that these two mechanisms, while conceptually distinct, jointly account for the majority of the total effect (60.0%–67.3%). This finding suggests that the impact of social media feedback on mental health is predominantly routed through self-evaluative processes—that is, the psychological harm associated with low feedback stems not from the absence of feedback per se, but from what low feedback implies about one’s social worth (via the sociometer) and relative standing (via social comparison). This self-evaluation framework offers a parsimonious theoretical account that can integrate findings from diverse research traditions on social media and mental health. It also carries a corollary implication: interventions that strengthen individuals’ capacity to decouple their self-evaluation from external digital metrics may be more effective than interventions that merely reduce social media exposure time.

Finally, it is important to acknowledge the theoretical boundaries of the present framework. The current model assumes a broadly linear relationship between feedback frequency and psychological outcomes—that is, more feedback is associated with better outcomes. However, this assumption may not hold across the full range of feedback experiences. The assumption of linearity warrants critical examination from multiple theoretical angles. First, from the perspective of self-determination theory [45], self-esteem that is contingent upon external validation—such as receiving likes and comments—may be qualitatively different from autonomous, stable self-esteem grounded in intrinsic self-worth. Crocker and Wolfe (2001) [46] proposed the concept of contingent self-worth, arguing that individuals who base their self-esteem heavily on external sources of approval develop a form of self-esteem that is inherently fragile and unstable, fluctuating in response to the presence or absence of validating feedback. Applying this framework to the social media context, individuals who consistently receive high levels of positive feedback may come to depend on this feedback as a primary source of self-validation. While this dependency may sustain elevated self-esteem in the short term, it simultaneously renders self-esteem increasingly vulnerable to any future reduction or fluctuation in feedback, potentially creating a psychological profile that is more—rather than less—susceptible to emotional distress over time.

Second, the reinforcement mechanisms inherent in social media platforms may transform high feedback into a driver of problematic usage patterns. Drawing on the reinforcement sensitivity framework [47], receiving likes and comments activates reward-related neural circuits, producing immediate positive affect that reinforces continued engagement. For individuals who habitually receive high levels of feedback, this positive reinforcement may establish a behavioral pattern akin to a variable-ratio reinforcement schedule—the same mechanism that underlies gambling and other addictive behaviors—wherein users repeatedly check and post content in anticipation of social rewards. Over time, this cycle may escalate into compulsive social media use, characterized by increasing time investment, difficulty disengaging, and anxiety when unable to access platforms. Paradoxically, the very feedback that initially supported positive self-evaluation may thus become a catalyst for behavioral patterns that ultimately undermine well-being.

Third, these considerations collectively suggest that the relationship between social media feedback and psychological outcomes may follow a nonlinear, potentially curvilinear pattern rather than the linear model tested in the present study. At low levels, increases in feedback may be genuinely protective, providing necessary signals of social acceptance and belonging. Beyond a certain threshold, however, additional feedback may yield diminishing psychological returns as individuals become habituated to high feedback levels, and may eventually become detrimental if it fosters contingent self-worth and compulsive feedback-seeking behaviors. This theoretical possibility aligns with the broader “Goldilocks hypothesis” proposed in screen time research [48], which suggests that moderate levels of digital engagement are associated with the best outcomes, while both low and excessive levels are associated with poorer well-being.

The present study, by design, tested only linear associations and therefore cannot empirically adjudicate between linear and curvilinear models. This represents an important theoretical boundary of our framework. Future research should explicitly test nonlinear effects—for example, by including quadratic terms for feedback frequency in regression models or by using person-centered approaches (e.g., latent profile analysis) to identify subgroups of users with distinct feedback-wellbeing profiles. Additionally, longitudinal studies that track the development of feedback dependency over time, incorporating measures of contingent self-worth and problematic social media use as additional variables, would help elucidate whether and under what conditions high feedback transitions from being psychologically beneficial to potentially harmful.

### Implications, limitations and conclusions

At the theoretical level, this study integrates sociometer theory and social comparison theory, constructing a dual-mediation longitudinal model that expands understanding of the internal mechanisms through which social media influences mental health. At the practical level, research findings suggest that mental health education targeting college students can incorporate social media literacy into curriculum content, helping young users recognize that like and comment counts are not valid indicators of personal worth. University psychological counseling services should attend to students’ social media usage patterns, particularly for individuals with low self-esteem or high social comparison tendencies [19]. Design changes such as Instagram’s initiative to hide like counts can also receive empirical support from this study [14].

Several limitations of this study should be considered when interpreting results. Although the longitudinal design and cross-lagged panel modeling approach establish temporal precedence and control for autoregressive effects, this study remains observational in nature. Causal conclusions cannot be drawn, as the possibility of unmeasured confounding variables—such as personality traits, life events, or peer relationship quality—cannot be ruled out. Experimental or quasi-experimental designs would be needed to establish definitive causal relationships between social media feedback and mental health outcomes. The sample was drawn from three universities in a provincial capital city in China, which may limit cross-cultural generalization of conclusions. Furthermore, participant recruitment was conducted primarily through online platforms and social media channels, which may have led to an overrepresentation of digitally active users. The inclusion criterion requiring participants to post social media content at least once per week further excluded low-frequency users. As a result, the present findings may be more applicable to regular, active social media users rather than to the full spectrum of young adults, including those who primarily consume content passively without posting. Future research should employ more diverse sampling strategies to include users with varying levels of social media engagement. All variables were measured through self-report, which introduces several potential biases. Most notably, the measurement of social media feedback frequency relied on participants’ retrospective recall of how often their content received likes and comments over the past month. Such retrospective self-reports are susceptible to recall bias, as participants may not accurately remember the actual frequency of feedback received. Moreover, recall accuracy may be systematically influenced by current emotional states—for example, individuals experiencing depressive symptoms may underestimate the amount of positive feedback they have received, which could inflate the observed association between low feedback and depression. Future research should consider integrating objective behavioral data—such as actual like and comment counts retrieved through platform APIs or screen-tracking applications—or employing experience sampling methods that capture feedback perceptions in real time, thereby reducing the reliance on retrospective recall and improving measurement accuracy. Additionally, self-esteem and social comparison were both measured at the same time point (T2) and modeled as parallel mediators, which prevented examination of potential serial mediation pathways between them. Future longitudinal research with additional measurement waves could test whether these mediators operate in a specific causal sequence, such as whether social comparison precedes changes in self-esteem or vice versa. The study did not distinguish between different social media platforms, nor did it differentiate the emotional valence of comments received. The feedback measures used in this study focused exclusively on the frequency of positive feedback, while negative comments—such as critical remarks, hostile responses, or cyberbullying—were not assessed. This is a notable omission, as negative comments may constitute a more potent and direct risk factor for psychological distress than the mere absence of positive feedback. It is possible that the psychological impact of receiving explicitly negative comments operates through different mechanisms than those examined in the present study. Future research should separately measure positive and negative feedback and examine whether their effects on mental health are symmetric or whether negative feedback exerts disproportionately stronger effects, consistent with the well-established negativity bias in psychological processing [49]. Additionally, distinguishing between platforms with different design features—such as the semi-private nature of WeChat Moments versus the more public and comparison-oriented interface of Xiaohongshu—would help clarify whether platform architecture moderates the effects of social media feedback on mental health [31].

In summary, this study provides empirical evidence through a three-wave longitudinal design for the impact of social media feedback on depressive and anxiety symptoms among young adult users, and reveals the dual mediating roles of self-esteem and social comparison. The findings emphasize the importance of attending to psychological mediating mechanisms when examining the effects of social media on mental health. Enhancing self-esteem and cultivating healthy social comparison strategies may be effective approaches to mitigate the negative psychological effects of social media use.

## Conclusions

Through an eight-month, three-wave longitudinal tracking survey of 398 Chinese young adult social media users, this study systematically examined the effects of social media likes and comments feedback on depressive and anxiety symptoms and their underlying psychological mechanisms. Results demonstrated that lower frequency of positive social media feedback significantly predicted higher levels of depressive and anxiety symptoms at subsequent time points, while the reverse paths were not established, providing longitudinal evidence supporting the temporal directionality of the relationship between social media feedback and mental health. Self-esteem and social comparison tendency played important mediating roles in these relationships, together explaining 65.0% of the total effect, indicating that social media feedback primarily affects emotional health status by influencing individuals’ self-evaluation systems.

The theoretical contribution of this study lies in integrating sociometer theory and social comparison theory to construct a dual-mediation longitudinal model of social media feedback’s influence on mental health, deepening understanding of the formation mechanisms of mental health issues among young adults in digitized social contexts. At the practical level, research findings suggest that mental health interventions targeting young populations can proceed in two directions: enhancing self-esteem resilience and cultivating healthy social comparison strategies, helping young users establish rational cognition regarding social media feedback and reducing the tendency to use like and comment counts as standards for self-worth evaluation. University mental health education and counseling services should incorporate social media literacy into their work content. Social media platforms can also reduce potential negative effects on user mental health by decreasing the visibility and comparability of feedback data. Future research can validate the present findings across broader cultural contexts and populations, and further distinguish the differential effects of different platform characteristics and feedback types.

## Data Availability

The datasets used and/or analysed during the current study available from the corresponding author on reasonable request.

## References

[1] Burrow AL, Rainone N. How many likes did I get?: Purpose moderates links between positive social media feedback and self-esteem. J Exp Soc Psychol. 2017;69:232–6.

[2] Chua THH, Chang L. Follow me and like my beautiful selfies: Singapore teenage girls’ engagement in self-presentation and peer comparison on social media. Comput Hum Behav. 2016;55:190–7.

[3] Meng L, Chen Y, Chen DT, Li X, Ma B, Liu Y, et al. Problematic social media use and depressive outcomes among college students in China: observational and experimental findings. Int J Environ Res Public Health. 2022;19(9):4937. 35564330 10.3390/ijerph19094937 PMC9099455

[4] Zhou W, Yan Z, Yang Z, Hussain Z. Problematic social media use and mental health risks among first-year Chinese undergraduates: a three-wave longitudinal study. Front Psychiatry. 2023;14:1237924. 37743982 10.3389/fpsyt.2023.1237924 PMC10512716

[5] Hayes RA, Carr CT, Wohn DY. One click, many meanings: interpreting paralinguistic digital affordances in social media. J Broadcast Electron Media. 2016;60(1):171–87.

[6] Scissors L, Burke M, Wengrovitz S. What’s in a Like? Attitudes and behaviors around receiving Likes on Facebook. In: Proceedings of the 19th ACM Conference on Computer-Supported Cooperative Work & Social Computing. New York: ACM; 2016. pp. 1501–10.

[7] Huang C. Time spent on social network sites and psychological well-being: a meta-analysis. Cyberpsychol Behav Soc Netw. 2017;20(6):346–54. 28622031 10.1089/cyber.2016.0758

[8] Huang C. A meta-analysis of the problematic social media use and mental health. Int J Soc Psychiatry. 2022;68(1):12–33. 33295241 10.1177/0020764020978434

[9] Shannon H, Bush K, Villeneuve PJ, Hellemans KGC, Guimond S. Problematic social media use in adolescents and young adults: systematic review and meta-analysis. JMIR Ment Health. 2022;9(4):e33450. 35436240 10.2196/33450 PMC9052033

[10] Thomas HJ, Dykxhoorn J, Power E, Ford T. Social media use and internalizing symptoms in clinical and community adolescent samples: a systematic review and meta-analysis. JAMA Pediatr. 2024;178(8):789–97. 10.1001/jamapediatrics.2024.2078 PMC11197453 38913335

[11] Twenge JM, Haidt J, Lozano J, Cummins KM. Specification curve analysis shows that social media use is linked to poor mental health, especially among girls. Acta Psychol. 2022;224:103512. 10.1016/j.actpsy.2022.103512 35101738

[12] Nesi J, Choukas-Bradley S, Prinstein MJ. Transformation of adolescent peer relations in the social media context: Part 1—a theoretical framework and application to dyadic peer relationships. Clin Child Fam Psychol Rev. 2018;21(3):267–94. 29627907 10.1007/s10567-018-0261-x PMC6435354

[13] Nesi J, Prinstein MJ. Using social media for social comparison and feedback-seeking: gender and popularity moderate associations with depressive symptoms. J Abnorm Child Psychol. 2015;43(8):1427–38. 25899879 10.1007/s10802-015-0020-0 PMC5985443

[14] Rodgers RF, McLean SA, Paxton SJ. Social media usage and body image: examining the mediating roles of internalization of appearance ideals and social comparisons in young women. Comput Human Behav. 2022;135:107290.

[15] Stefana A, Dakanalis A, Lavalle C, Matera C, Nerini A. Instagram use and mental well-being: The mediating role of social comparison. J Nerv Ment Dis. 2023;211(1):47–54. 10.1097/NMD.0000000000001577 36449721

[16] Plackett R, Sheringham J, Dykxhoorn J. The longitudinal impact of social media use on UK adolescents’ mental health: longitudinal observational study. J Med Internet Res. 2023;25:e43213. 36961482 10.2196/43213 PMC10132039

[17] Irmer A, Schmiedek F. Associations between youth’s daily social media use and well-being are mediated by upward comparisons. Commun Psychol. 2023;1:12. 39242889 10.1038/s44271-023-00013-0 PMC11332017

[18] Keles B, McCrae N, Grealish A. A systematic review: The influence of social media on depression, anxiety and psychological distress in adolescents. Int J Adolesc Youth. 2020;25(1):79–93.

[19] Zhang H, Li Z, Huang Y, Chen Y, Pang H. Does WeChat use intensity influence Chinese college students’ mental health through social use of WeChat, entertainment use of WeChat, and bonding social capital? Front Public Health. 2023;11:1167172. 38074739 10.3389/fpubh.2023.1167172 PMC10704145

[20] Markus HR, Kitayama S. Culture and the self: implications for cognition, emotion, and motivation. Psychol Rev. 1991;98(2):224–53.

[21] Qi X. Face: a Chinese concept in a global sociology. J Sociol. 2011;47(3):279–95.

[22] Leary MR, Tambor ES, Terdal SK, Downs DL. Self-esteem as an interpersonal monitor: the sociometer hypothesis. J Pers Soc Psychol. 1995;68(3):518–30.

[23] Ozimek P, Bierhoff HW. All my online-friends are better than me—Three studies about ability-based comparative social media use, self-esteem, and depressive tendencies. Behav Inf Technol. 2020;39(10):1110–23.

[24] Jiang S, Ngien A. The effects of Instagram use, social comparison, and self-esteem on social anxiety: a survey study in Singapore. Social Media + Society. 2020;6(2):2056305120912488. 10.1177/2056305120912488.

[25] Valkenburg PM, Beyens I, Pouwels JL, van Driel II, Keijsers L. Social media use and adolescents’ self-esteem: heading for a person-specific media effects paradigm. J Commun. 2021;71(1):56–78.

[26] Valkenburg PM, Pouwels JL, Beyens I, van Driel II, Keijsers L. Adolescents' social media experiences and their self-esteem: a person-specific susceptibility perspective. Technology, Mind, and Behavior. 2021;2(2). 10.1037/tmb0000037.

[27] Valkenburg PM, Peter J. The differential susceptibility to media effects model. J Commun. 2013;63(2):221–43.

[28] Festinger L. A theory of social comparison processes. Human Relations. 1954;7(2):117–40.

[29] Coyne SM, Rogers AA, Zurcher JD, Stockdale L, Booth M. Does time spent using social media impact mental health?: An eight year longitudinal study. Comput Human Behav. 2020;104:106160.

[30] Ozimek P, Bierhoff G, Rohmann E, Bierhoff HW. The impact of social comparisons more related to ability vs. more related to opinion on well-being: An Instagram study. Behav Sci. 2023;13(10):850. 37887500 10.3390/bs13100850 PMC10604111

[31] Chao M, Lei J, He R, Jiang Y, Yang H. TikTok use and psychosocial factors among adolescents: Comparisons of non-users, moderate users, and addictive users. Psychiatry Res. 2023;325:115247. 37167877 10.1016/j.psychres.2023.115247

[32] Jiang C, Zhu Y, Luo Y, Meng R, Costa DJ, Costa AR. Validation of the Chinese version of the Rosenberg Self-Esteem Scale: Evidence from a three-wave longitudinal study. BMC Psychol. 2023;11(1):345. 37853499 10.1186/s40359-023-01293-1 PMC10585735

[33] Gibbons FX, Buunk BP. Individual differences in social comparison: Development of a scale of social comparison orientation. J Pers Soc Psychol. 1999;76(1):129–42. 9972558 10.1037//0022-3514.76.1.129

[34] Wang W, Bian Q, Zhao Y, Li X, Wang W, Du J, et al. Reliability and validity of the Chinese version of the Patient Health Questionnaire (PHQ-9) in the general population. Gen Hosp Psychiatry. 2014;36(5):539–44. 25023953 10.1016/j.genhosppsych.2014.05.021

[35] Tong X, An D, McGonigal A, Park SP, Zhou D. Validation of the Generalized Anxiety Disorder-7 (GAD-7) among Chinese people with epilepsy. Epilepsy Res. 2016;120:31–6. 26709880 10.1016/j.eplepsyres.2015.11.019

[36] Hamaker EL, Kuiper RM, Grasman RPPP. A critique of the cross-lagged panel model. Psychol Methods. 2015;20(1):102–16. 25822208 10.1037/a0038889

[37] Maxwell SE, Cole DA. Bias in cross-sectional analyses of longitudinal mediation. Psychol Methods. 2007;12(1):23–44. 17402810 10.1037/1082-989X.12.1.23

[38] Vogel EA, Rose JP, Roberts LR, Eckles K. Social comparison, social media, and self-esteem. Psychol Pop Media Cult. 2014;3(4):206–22.

[39] Mulder JD, Hamaker EL. Three extensions of the random intercept cross-lagged panel model. Struct Equ Modeling. 2021;28(4):638–48.

[40] Cohen J. Statistical power analysis for the behavioral sciences. 2nd ed. Lawrence Erlbaum Associates; 1988.

[41] Funder DC, Ozer DJ. Evaluating effect size in psychological research: Sense and nonsense. Adv Methods Pract Psychol Sci. 2019;2(2):156–68.

[42] American Psychiatric Association. Diagnostic and statistical manual of mental disorders. 5th ed. Arlington, VA: American Psychiatric Publishing; 2013.

[43] Forest AL, Wood JV. When social networking is not working: Individuals with low self-esteem recognize but do not reap the benefits of self-disclosure on Facebook. Psychol Sci. 2012;23(3):295–302. 22318997 10.1177/0956797611429709

[44] Dormann C, Griffin MA. Optimal time lags in panel studies. Psychol Methods. 2015;20(4):489–505. 26322999 10.1037/met0000041

[45] Deci EL, Ryan RM. The “what” and “why” of goal pursuits: human needs and the self-determination of behavior. Psychol Inq. 2000;11(4):227–68.

[46] Crocker J, Wolfe CT. Contingencies of self-worth. Psychol Rev. 2001;108(3):593–623. 11488379 10.1037/0033-295x.108.3.593

[47] Turel O, Serenko A. The benefits and dangers of enjoyment with social networking websites. Eur J Inf Syst. 2012;21(5):512–28.

[48] Przybylski AK, Weinstein N. A large-scale test of the Goldilocks hypothesis: quantifying the relations between digital-screen use and the mental well-being of adolescents. Psychol Sci. 2017;28(2):204–15. 28085574 10.1177/0956797616678438

[49] Baumeister RF, Bratslavsky E, Finkenauer C, Vohs KD. Bad is stronger than good. Rev Gen Psychol. 2001;5(4):323–70.

